# Editorial for the Special Issue on MEMS/NEMS Devices and Applications, 3rd Edition

**DOI:** 10.3390/mi17020205

**Published:** 2026-02-02

**Authors:** Zhi-Xuan Dai, Takahito Ono, Ching-Liang Dai

**Affiliations:** 1 Department of Bio-Industrial Mechatronics Engineering, National Chung Hsing University, Taichung 402, Taiwan; 2 Department of Mechanical Systems Engineering, Tohoku University, Sendai 980-8579, Japan; 3 Department of Mechanical Engineering, National Chung Hsing University, Taichung 402, Taiwan

## 1. Introduction

Microelectromechanical systems (MEMS) and nanoelectromechanical systems (NEMS) have experienced rapid and sustained development in recent years and have become key enabling technologies for intelligent sensing [1,2,3,4], energy harvesting [5,6,7,8,9,10], biomedical devices [11], and Internet of Things (IoT) [12] applications. A wide variety of MEMS/NEMS devices have already been developed and successfully deployed in practical systems. For example, capacitive accelerometers are extensively used for motion sensing in smartphones, wearable electronics, automotive safety systems, and structural or vibration monitoring, as their proof mass–spring architectures convert acceleration into measurable capacitance variations with excellent scalability [13]. Vibrating gyroscopes complement accelerometers in inertial measurement units and play a critical role in camera stabilization, navigation, robotics, and vehicle stability control by providing accurate angular-rate information in a compact, low-power form [14]. Pressure sensors, including capacitive, piezoresistive, and emerging resonant types, are widely applied in tire-pressure monitoring, medical catheters, respiratory equipment, and industrial process control, where advances in materials and packaging continue to enhance sensitivity and long-term reliability [15]. MEMS microphones are now the standard audio interface in smartphones, smart speakers, and wearable devices, supporting voice recognition, noise cancellation, and beamforming with high sensitivity and a small form factor [16]. Infrared sensors are extensively applied in thermal imaging for night vision, firefighting, industrial inspection, and human presence detection by converting infrared radiation into electrical signals [17]. For reconfigurable radio-frequency systems, RF MEMS switches offer low insertion loss, high isolation, and near-zero static power consumption, supporting tunable filters, phase shifters, and reconfigurable antennas, particularly in satellite and space-constrained communication platforms [18]. In three-dimensional perception, scanning micromirrors enable compact LiDAR beam steering for autonomous vehicles, drones, and robotic systems, offering reduced size, high scanning speed, and cost advantages over conventional mechanical scanners [19]. In minimally invasive healthcare, microneedle arrays create micron-scale pathways through the stratum corneum, enabling painless transdermal drug and vaccine delivery as well as biofluid sampling for point-of-care diagnostics and theranostic patches [20]. Gas sensors, especially metal–oxide-based sensors integrated with microheaters, are used for environmental monitoring, industrial safety, and indoor air-quality control, enabling rapid and selective detection of hazardous gases at low power consumption [21]. Surface acoustic wave (SAW) sensors serve as highly sensitive platforms for chemical and biosensing, where functionalized piezoelectric substrates and interdigitated transducers convert mass loading or viscoelastic changes into measurable frequency or phase shifts [22]. In lab-on-a-chip automation, microfluidic membrane valves enable reliable on-chip flow routing, metering, and isolation, facilitating portable analytical systems and integrated sample preparation without bulky external equipment [23]. Finally, MEMS microgrippers support precise micromanipulation in microassembly and biomedical handling of cells, microtissues, and delicate micro-objects, where compliant mechanisms and compact actuation allow accurate pick-and-place operations and gentle gripping at the microscale [24].

**Figure 1:**
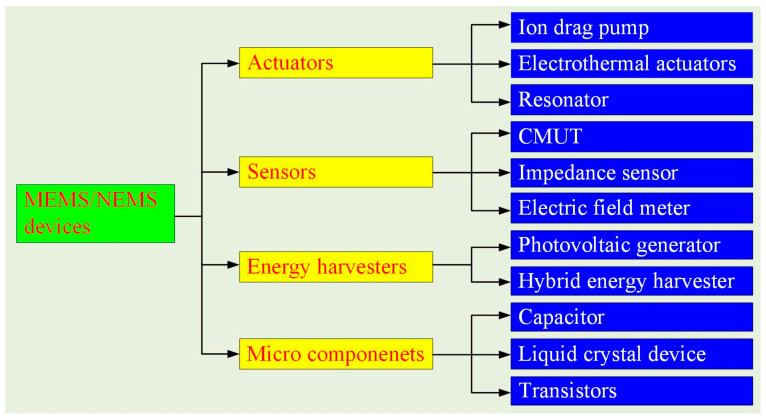
Classification of MEMS/NEMS devices presented in this Special Issue.

In the previous 2nd Edition Special Issue, a broad range of NEMS/MEMS devices was reported [25], covering diverse functional categories such as actuators [26,27,28,29], antennas [30], sensors [31,32,33,34,35], and energy harvesters [36], reflecting the rapid expansion of micro- and nano-scale system technologies. Building upon this foundation, the present Special Issue further consolidates recent advances in MEMS/NEMS device design, fabrication innovation, sensors, actuators, and microscale components, highlighting both fundamental developments and application-oriented progress. As illustrated in Figure 1, the contributions in this Special Issue can be systematically classified according to device functionality. In the actuator category, reported works include a light-controlled ion drag pump [Contribution 1], electrothermal actuators [Contribution 2], a high-frequency resonator [Contribution 3], and electrothermal co-actuation devices [Contribution 4], demonstrating diverse actuation mechanisms and structural strategies. The sensor-related studies encompass a capacitive micromachined ultrasonic transducer (CMUT) [Contribution 5], an impedance microsensor [Contribution 6], and a DC electric field meter [Contribution 7], addressing challenges in precision sensing, robustness, and environmental adaptability. In the area of energy harvesting, this Special Issue features a CMOS-compatible photovoltaic generator [Contribution 8] and a rotary piezoelectric–electromagnetic hybrid energy harvester [Contribution 9], emphasizing self-powered operation and efficient energy conversion. Additionally, several key microcomponents are presented, including stacked metal–insulator–metal capacitors [Contribution 10], an electro-optic liquid crystal device [Contribution 11], a P-channel vertical double-diffused metal–oxide–semiconductor (VDMOS) transistor [Contribution 12], and a tunnel field-effect transistor [Contribution 13], which collectively underscore ongoing progress in microelectronic technology.

## 2. MEMS/NEMS Actuators in Special Issue

Tang et al. [Contribution 1] report an experimental investigation of a light-controlled ion drag pump that eliminates conventional wired high-voltage supplies by exploiting the photoelectric properties of lanthanum-modified lead zirconate titanate (PLZT) ceramics. The central research theme is to demonstrate how optically generated high photovoltage can be directly coupled with electrohydrodynamic (EHD) charge injection to realize wireless, non-contact fluid transport. The main innovation lies in integrating the anomalous photovoltaic effect of PLZT with an ion drag pumping mechanism, enabling kilovolt-level driving voltages under ultraviolet illumination without external power conditioning, which fundamentally differs from traditional EHD pumps relying on bulky, noise-prone high-voltage sources. Methodologically, the study combines theoretical modeling, numerical analysis, and systematic experiments to clarify the relationships among photovoltage generation, electric field formation, charge injection, and fluid motion. The device consists of a PLZT ceramic patch serving as a light-activated voltage source and a multilayer pump chip composed of a dielectric substrate, patterned serrated electrodes, a microfluidic channel, and a cover layer. Under UV irradiation, the PLZT generates a rapidly rising photovoltage that drives field emission at the emitter electrodes, forming ions within a dielectric fluid; the resulting Coulomb force transports the fluid toward the collector electrodes. The fabrication approach emphasizes relatively simple planar electrode patterning and dielectric channel assembly, ensuring electrical insulation and preventing breakdown while maintaining compatibility with microfabrication techniques. Experimentally, key structural parameters, which include channel height, electrode spacing, number of electrode pairs, and inter-pair gaps, are varied to quantify their effects on response time, flow rate, and output pressure. The results show that higher light intensity accelerates activation and enhances performance, while reduced electrode spacing and increased electrode pair numbers significantly improve pressure and flow. An optimal electrode gap is identified that balances opposing electric fields and hydrodynamic losses. Importantly, the pump achieves flow rates on the order of hundreds of microliters per minute and stall pressures approaching 1 kPa under purely optical control. Overall, the work establishes a new light-driven pumping paradigm with low electromagnetic interference and strong spatial controllability, offering valuable design guidelines for future applications in microfluidics, soft robotics, and optically addressable microsystems.

Tang et al. [Contribution 2] explore the comprehensive electrothermal–thermomechanical analysis of a V-shaped micro electrothermal actuator, aiming to establish an accurate and efficient numerical framework for predicting its driving behavior under electrical excitation. The primary innovation of the work lies in the introduction of the polynomial point interpolation collocation method (PPCM) as a strong-form, meshfree computational approach to solve the highly coupled, nonlinear multiphysics problem inherent in electrothermal actuation. Unlike conventional finite-element methods that rely on meshing and may suffer from remeshing or numerical instability, the proposed PPCM directly constructs local polynomial-based interpolation functions at discrete nodes, enabling stable and efficient analysis for microscale structures. The research methodology integrates electrothermal modeling with thermomechanical deformation analysis in a sequential yet fully coupled manner. First, the temperature field generated by Joule heating is obtained by solving nonlinear heat transfer equations that account for temperature-dependent electrical resistivity and thermal conductivity. Subsequently, the resulting temperature distribution is incorporated into a thermoelastic model to predict actuator displacement. The actuator is fabricated from polysilicon and adopts a planar V-shaped beam configuration, a geometry well known for amplifying lateral displacement through asymmetric thermal expansion. The structural dimensions, material properties, and boundary conditions are defined to reflect realistic MEMS fabrication constraints, while the numerical formulation avoids excessive geometric simplifications. An incremental load strategy is embedded into the PPCM to enhance convergence when handling strong material nonlinearities. To validate the proposed approach, numerical predictions of temperature and displacement are systematically compared with both finite-element simulations and experimental measurements obtained from infrared thermal microscopy and prior mechanical characterization. The results demonstrate that the PPCM achieves temperature predictions within approximately 10% of experimental values and displacement errors as low as 2 μm at higher driving voltages, closely matching FEM results. Overall, this study confirms that the PPCM provides a robust, accurate, and computationally efficient tool for analyzing electrothermal microactuators, offering significant potential for future MEMS design optimization and multiphysics simulation without the limitations of traditional mesh-based methods.

Lin et al. [Contribution 3] investigate a high-frequency film bulk acoustic resonator (FBAR) operating near 6.5 GHz, with the primary objective of mitigating lateral acoustic wave leakage that severely limits the quality factor of conventional high-frequency resonators. The core innovation is the introduction of a Mo/SiC composite microstructure strategically embedded along the boundary of the active resonant region to create multiple acoustic impedance discontinuities, thereby reflecting laterally propagating acoustic waves back into the resonator. Unlike prior approaches that rely mainly on longitudinal Bragg reflectors or electrode-based frames, this work exploits the strong acoustic impedance contrast between molybdenum and silicon carbide to enhance in-plane energy confinement without compromising structural integrity. The research methodology integrates acoustic impedance theory, finite-element multiphysics simulations, and experimental validation to systematically optimize microstructure thickness and width while suppressing spurious resonance modes. Structurally, the device adopts a sandwich configuration consisting of Mo top and bottom electrodes, a Sc_0.2_Al_0.8_N piezoelectric film, and an air cavity, fabricated on a SiCOI substrate to ensure high crystalline quality of the piezoelectric layer. The Mo/SiC composite microstructures are laterally distributed near the resonator edge, with carefully optimized sectional widths to balance acoustic reflection and mode purity. Fabrication is realized through an advanced thin-film bonding and transfer process, combined with sputtering, dry etching, wafer bonding, chemical–mechanical polishing, and XeF_2_ cavity release, enabling precise control of multilayer thickness and microstructure geometry. Experimental radio-frequency (RF) measurements confirm that the optimized resonator achieves a series resonance frequency of 6.488 GHz with a parallel-mode quality factor of 310, representing a 51.2% improvement over the baseline FBAR while maintaining acceptable electromechanical coupling. Statistical measurements across multiple devices further demonstrate good repeatability and stability. Overall, this work provides both a practical fabrication route and a robust structural design strategy for suppressing lateral acoustic losses, offering important guidance for the development of next-generation low-loss RF resonators for advanced wireless communication systems.

Tang et al. [Contribution 4] investigate a multi-electrothermal co-actuation device for safety-and-arming mechanisms in loitering munition fuzes, targeting reliable large displacement under extreme overload. The main contribution is a co-actuation architecture that integrates multiple V-shaped electrothermal actuators, where primary units generate displacement and auxiliary units provide position holding and bidirectional control for improved robustness. An electrothermal–mechanical coupled model is established and validated through finite-element simulations and extensive experiments. The actuator beams use stainless steel 304 to enhance strength and overload tolerance, and the structure is fabricated by laser processing to precisely pattern thick metal components. Key parameters, including V-beam number, air-gap thickness, contact type, load, driving voltage waveform, heat transfer, and gas damping, are systematically evaluated. Results show that optimized point–contact interfaces and multi-beam designs increase displacement efficiency, while appropriate thermal management reduces performance degradation. High-overload impact tests further demonstrate stable operation beyond 12,000 g without fracture, with only minor recoverable deformation. Overall, the proposed strategy delivers both enhanced electrothermal actuation and exceptional mechanical resilience for high-reliability micro-actuation applications.

## 3. MEMS/NEMS Sensors in Special Issue

He et al. [Contribution 5] report the development of a CMUT-based linear array system for non-contact thickness measurement of marine engineering structures under varying environmental conditions. The work overcomes the limitations of conventional piezoelectric ultrasonic transducers in underwater environments by exploiting MEMS-based CMUT technology, which provides wide bandwidth, low power consumption, and improved acoustic impedance matching with water. The key contribution is the system-level integration of a high-density 16-element CMUT linear array with polyurethane encapsulation and customized transmit–receive electronics, enabling stable and accurate thickness measurements without physical contact. The study combines analytical modeling of CMUT electromechanical behavior, membrane-based structural design, MEMS fabrication, and comprehensive experimental validation. Each CMUT element comprises numerous micro-capacitive cells formed by a single-crystal silicon membrane, a vacuum cavity, and insulated electrodes, allowing efficient bidirectional acoustic–electrical transduction. The array is fabricated using silicon–silicon wafer bonding, followed by cavity etching, wafer-level bonding, substrate thinning, membrane release, dielectric deposition, and metal sputtering to ensure uniform membranes and high yield. Polyurethane encapsulation is applied to provide electrical insulation and environmental protection while minimizing acoustic reflection losses due to its water-like impedance. The complete system integrates high-voltage pulse excitation and low-noise signal reception, with thickness determined using a time-of-flight algorithm enhanced by signal processing techniques. Experimental results show a transmitting sensitivity of 146.82 dB and a receiving sensitivity of −229.55 dB at 1 MHz. Importantly, tests across different water temperatures and salinity levels demonstrate thickness errors within ±0.1 mm, confirming the robustness and reliability of the proposed system for marine applications.

Shi et al. [Contribution 6] propose a novel debris material identification approach based on impedance microsensing, targeting the long-standing challenge of accurately distinguishing metal wear particles in lubricating oil for machinery condition monitoring. Instead of relying solely on optical appearance or inductive amplitude, the study introduces an impedance-based strategy that jointly exploits variations in inductance and alternating-current resistance induced by metal debris passing through a micro-coil sensor. The key innovation is recognizing that different metals exhibit unique combinations of magnetic permeability and electrical conductivity, which lead to distinct, nonlinear inductance–resistance amplitude trends as particle size increases. To validate this concept, the authors combine electromagnetic theory, finite-element simulations, and controlled experiments to establish a database of characteristic amplitude–size curves for multiple metals. The sensing device consists of a coil-based impedance microsensor integrated with a microfluidic channel of 900 µm diameter, where a solenoid coil with precisely defined geometry surrounds the flow path to ensure stable and repeatable electromagnetic excitation. The sensor is fabricated using a casting-based process, including coil winding, mold formation, substrate curing, and channel construction, enabling a compact and robust structure suitable for inline oil monitoring. During operation, metal particles driven by a syringe pump traverse the sensing zone under a high-frequency excitation, and the resulting impedance variations are captured using precision instrumentation and signal processing techniques such as filtering and baseline correction. Experimental results demonstrate that ferromagnetic particles generate positive inductance pulses, while non-ferromagnetic particles produce negative inductance responses, whereas all metals increase AC resistance to different extents. By jointly analyzing inductance and resistance signatures and comparing them with the pre-established database, the system successfully distinguishes iron, stainless steel, aluminum, copper, and brass particles, even when colors are similar or surfaces are contaminated. Importantly, the method is insensitive to oil transparency, impurities, and surface dirt, overcoming key limitations of optical techniques. Overall, the study delivers a robust, physics-driven solution that enriches debris information beyond size and count, offering improved reliability for early fault diagnosis and advancing the practical capability of intelligent oil condition monitoring systems.

Yang et al. [Contribution 7] report the design, fabrication, and validation of a high-sensitivity three-dimensional DC electric field meter (3D EFM) based on MEMS technology, aiming to overcome the limited sensitivity, humidity susceptibility, and structural instability of previously reported MEMS EFMs. The core research focus is to realize accurate vector electric field measurement under real-world environmental conditions while maintaining compactness and low power consumption. The main innovation lies in a highly sensitive packaging strategy employing an inward-convex metallic cover, which significantly amplifies the electric field intensity within the sensor cavity compared with conventional flat-cover packages, as confirmed through analytical modeling and finite-element simulations. In addition, the study introduces a mechanically robust 1D electric field sensing chip (EFSC) architecture that replaces long flexible beams with short strip-type electrodes anchored on rigid frames, thereby improving vibration stability during resonant operation. The research methodology integrates theoretical electrostatic analysis, COMSOL-based multiphysics simulations, MEMS fabrication, package-level optimization, and full-system experimental calibration. Each EFSC is fabricated using a silicon-on-insulator process and operates in a lateral resonant vibration mode at approximately 3 kHz, driven by low-voltage electrostatic actuation. Three identically packaged EFSCs are incorporated into a cubic 3D EFM structure, where orthogonally arranged detecting electrodes couple the external electric field into the sensor packages. A moistureproof system-level enclosure, consisting of grounded metal chambers, Teflon insulators, tinplate shielding, and semi-rigid coaxial cables, is implemented to suppress humidity-induced polarization and ground potential interference. Wireless data transmission is further adopted to isolate the sensing unit from installation-dependent grounding effects. Experimental results demonstrate that the inward-convex package improves single-axis sensitivity from 1.95 to 7.19 mV/(kV/m), while the complete 3D EFM achieves a sensitivity at least 4.64 times higher than previously reported MEMS-based 3D EFMs. The maximum relative error under arbitrary orientation is limited to 2.2%, linearity remains within 1% even at high humidity, and the resolution of each axis is better than 10 V/m. These results verify that the proposed device offers a robust and practical solution for high-precision DC electric field vector measurement in demanding environments.

## 4. MEMS/NEMS Energy Harvesters in Special Issue

Chen et al. [Contribution 8] present the design and experimental verification of a CMOS-compatible micro photovoltaic generator (MPG) for compact, self-powered microsystems and IoT applications. The study tackles the limited efficiency of silicon-based MPGs fabricated using standard CMOS processes, where optical shading, shallow junction depths, and dielectric passivation layers reduce photon absorption and carrier collection. The main contribution is a mesh-patterned p–n junction design combined with a simple post-CMOS oxide removal process, which enhances photocurrent generation without modifying the foundry CMOS flow. Compared with conventional interdigitated layouts, the grid-like p-type regions embedded in a lightly doped n-well increase junction perimeter density and shorten lateral carrier transport paths, thereby reducing recombination losses. TCAD simulations are employed to optimize device geometry, followed by fabrication in a commercial 0.18 μm CMOS process and systematic electrical characterization under controlled illumination. The MPG is realized entirely using standard CMOS materials, including a p-type substrate, n-wells, doped junctions, and aluminum interconnects, ensuring full process compatibility and scalability. After fabrication, a wet etching step removes the oxide layer above the active regions, allowing more efficient light penetration into the depletion zones. The device is wire-bonded and mounted on a printed circuit board for testing. Experimental results indicate that oxide removal increases the short-circuit current by about 20.1%. Under 1000 W/m^2^ illumination, the MPG achieves 0.49 V open-circuit voltage, 239 μA short-circuit current, and 90 μW maximum output power, corresponding to 12.9% efficiency. These results demonstrate a practical approach to high-performance CMOS-integrated photovoltaic power sources for autonomous microsystems.

Yao et al. [Contribution 9] present the design and experimental demonstration of a rotary piezoelectric–electromagnetic hybrid energy harvester aimed at efficiently capturing low-frequency rotational mechanical energy commonly found in natural and industrial environments. The central research theme is to overcome the low power density and narrow operating bandwidth of single-mechanism harvesters by integrating complementary transduction principles within a compact mechanical architecture. The key innovation is the incorporation of a planetary gear transmission system with a 2:1 ratio, which effectively amplifies the external excitation frequency before it is delivered to the energy harvesting modules, thereby enhancing electrical output under low-speed rotation. Methodologically, the study combines theoretical modeling, multiphysics simulation, prototype fabrication, and systematic experimental testing. Analytical models are established to describe the kinematics of the slider–crank mechanism, magnetic coupling forces, piezoelectric response, and electromagnetic induction, while COMSOL and ANSYS simulations are used to predict magnetic fields, cantilever deformation, voltage generation, and power output. Structurally, the device integrates four piezoelectric energy harvesters (PEHs) and four electromagnetic energy harvesters (EMHs) arranged symmetrically around the gear system. The PEHs consist of PZT-5H ceramic layers bonded to stainless-steel cantilever beams, each carrying a rectangular NdFeB magnet, whereas the EMHs employ copper coils and cylindrical NdFeB magnets undergoing linear reciprocating motion. Magnetic coupling between moving magnets excites both piezoelectric bending and electromagnetic flux variation without direct mechanical contact, improving durability and reliability. The prototype is fabricated using a combination of 3D printing for the mechanical frame, precision assembly of gears and bearings, epoxy bonding of piezoelectric elements, and coil winding, enabling rapid realization of a robust mesoscale device. Experimental results show that, under an excitation frequency of 7 Hz with eight magnets, the hybrid harvester delivers a combined output voltage of 10.4 V and an average power of 250 mW, with an overall energy conversion efficiency of approximately 67%. Practical demonstrations further confirm its capability to light 60 LEDs and continuously power a temperature–humidity sensor. These results highlight the effectiveness of frequency amplification and hybrid transduction in harvesting low-frequency rotational energy, offering a promising solution for self-powered sensing and distributed IoT applications.

## 5. MEMS/NEMS Micro Components in Special Issue

Choi et al. [Contribution 10] examine the radio-frequency characteristics of stacked metal–insulator–metal (MIM) capacitors fabricated using RF CMOS technology, aiming to establish an accurate modeling approach for multi-GHz circuit applications. The study focuses on understanding how vertically stacked MIM structures, commonly used to enhance capacitance density, introduce additional parasitic effects that are inadequately described by conventional single-layer capacitor models. The key contribution is the development of an improved equivalent RF circuit that separately accounts for electrode-dependent parasitic resistance and inductance in each stacked layer, as well as the extra series impedance from the bottom electrode. To extract intrinsic device parameters, the authors compare one-step and two-step de-embedding methods and demonstrate that combined open–short de-embedding is necessary for reliable RF characterization. The methodology integrates wideband S-parameter measurements from 500 MHz to 20 GHz with systematic de-embedding and circuit modeling validated through Y-parameter analysis. The stacked MIM capacitors are fabricated in a standard 0.18 μm CMOS process using two vertically connected TiN/SiNx/TiN capacitor units to double capacitance per unit area. Multiple capacitor sizes and dedicated open and short test structures are implemented to evaluate scaling and parasitic effects. Experimental results show that single-step de-embedding causes under- or overestimation of capacitance and resistance, whereas the proposed two-step approach yields consistent and physically meaningful values. The resulting modified model accurately reproduces capacitance, quality factor, and S- and Y-parameters up to 20 GHz, significantly outperforming conventional models. These findings provide practical guidance for designing compact, high-density capacitors in high-frequency RF CMOS-integrated circuits.

Basu et al. [Contribution 11] investigate a material-based approach to accelerate electro-optic switching in nematic liquid crystal (LC) devices by suppressing mobile ionic impurities using helical carbon nanotubes (hCNTs). The study addresses a long-standing issue in LC technology, where residual ions from materials and interfaces degrade dynamic performance by increasing rotational viscosity and causing flicker and image sticking. The main contribution is the introduction of an ultralow concentration of non-functionalized hCNTs as physical ion traps, exploiting their spring-like geometry to capture both positive and negative ions without altering LC chemistry or requiring complex purification. The research combines material preparation, transient ion current analysis, dielectric spectroscopy, rotational viscosity extraction, and optical switching measurements. Nematic LC E7 serves as the host medium, while hCNTs are uniformly dispersed using ethanol-assisted sonication. The devices employ a planar LC cell structure with indium tin oxide electrodes and rubbed polyimide alignment layers, enabling reproducible electro-optic characterization using standard LC assembly processes. Experimental results show that hCNT doping at 3.1 × 10^−3^ wt.% reduces free ion concentration by 60–70% across a wide temperature range, leading to a 17% reduction in rotational viscosity and enhanced dielectric anisotropy. Consequently, optical relaxation time is shortened by approximately 25% while maintaining stable textures. The study also identifies an optimal concentration window, as excessive hCNT loading causes aggregation and electrical shorting. Overall, the work demonstrates a simple and effective strategy for improving LC switching speed and reliability.

Danković et al. [Contribution 12] present a comprehensive review of negative bias temperature instability (NBTI) in commercial power p-channel VDMOS transistors, focusing on how device-level degradation affects practical circuit performance. The review aims to bridge fundamental NBTI reliability studies and real application behavior by jointly considering threshold voltage shifts, self-heating, and circuit-level degradation. Instead of concentrating only on defect physics, the authors synthesize extensive long-term experimental results to demonstrate how NBTI-induced parameter changes degrade load-driving capability and logic operation. Key experimental methodologies are reviewed, including accelerated negative gate-bias stress at elevated temperatures, relaxation and annealing processes, and repeated electrical characterization of transfer characteristics. Special emphasis is placed on infrared thermography as an effective tool for visualizing and quantifying self-heating in VDMOS devices under realistic operating conditions. Circuit-level evaluation methods are also discussed, particularly CMOS inverter configurations incorporating stressed p-channel VDMOS transistors, which reveal changes in transfer curves, rise and fall times, and pulse narrowing effects. From a structural perspective, the review examines commercial p-channel VDMOS devices with polysilicon gates, thick gate oxides, and vertical double-diffused architectures packaged in TO-220 housings, highlighting their inherent susceptibility to NBTI and thermal stress due to high current capability. The consolidated findings show that NBTI leads to a progressive increase in absolute threshold voltage, higher on-resistance, and increased power dissipation, which further intensifies self-heating. This thermal feedback is identified as a critical reliability concern, especially at elevated temperatures. The review also notes that proper thermal management, such as heatsink use, can partially mitigate degradation, while challenges remain for long-term operation and complex circuit conditions.

Chen et al. [Contribution 13] survey the evolution and current state of tunnel field-effect transistors (TFETs) as a promising “Beyond Moore” solution for ultra-low-power electronics, focusing on how quantum-mechanical band-to-band tunneling can overcome the fundamental 60 mV/dec subthreshold swing limit of conventional MOSFETs. The central emphasis of the review is to systematically compare how material selection, device architectures, and fabrication strategies influence TFET switching steepness, on-state current, and leakage behavior, which are the key performance bottlenecks hindering large-scale adoption. Rather than presenting new device demonstrations, the article synthesizes a wide body of experimental and simulation-based studies, organizing them into coherent categories that reflect the historical and technological progression of TFET research. Experimentally reported devices based on Si, Si/Ge heterojunctions, III–V compound semiconductors, two-dimensional materials, and carbon nanotubes are critically examined, highlighting how bandgap size, band alignment, and effective mass determine tunneling efficiency. The review places particular weight on advanced device geometries—such as double-gate, gate-all-around nanowire, L-shaped, U-shaped, symmetric, and line-tunneling TFETs—that expand the tunneling area or enhance electrostatic control to simultaneously improve subthreshold swing and drive current. Complementing experimental results, a substantial portion of the review discusses TCAD-based simulation studies, which explore idealized architectures including dopingless, junctionless, negative-capacitance, heterogeneous-gate, and quasi-broken-gap TFETs, revealing the theoretical performance limits achievable through aggressive band and field engineering. Across these studies, the review identifies recurring methodological tools, including band-diagram analysis, tunneling probability modeling, and benchmarking using metrics such as minimum/average subthreshold swing, Ion/Ioff ratio, and operating voltage. The major conclusions drawn are that TFETs not only exhibit sub-60 mV/dec switching but, in optimized forms, can also achieve steep slopes below 10 mV/dec and competitive on-currents at supply voltages around 0.5 V, particularly when line tunneling or broken-gap heterojunctions are employed. Finally, the review outlines future challenges related to variability, fabrication complexity, and integration while emphasizing emerging application opportunities in ultra-low-power logic, biosensors, and energy-efficient integrated circuits, thereby providing a consolidated roadmap for both device engineers and circuit designers working toward next-generation low-power systems.
